# Effectiveness of tocotrienol-rich fraction combined with tamoxifen in the management of women with early breast cancer: a pilot clinical trial

**DOI:** 10.1186/bcr2726

**Published:** 2010-10-08

**Authors:** Kalanithi Nesaretnam, Kanga Rani Selvaduray, Ghazali Abdul Razak, Sheela Devi Veerasenan, Patricia A Gomez

**Affiliations:** 1 Malaysian Palm Oil Board, 6 Persiaran Institusi, Bandar Baru Bangi, Kajang, 43000 Selangor Darul Ehsan, Malaysia; 2 Kuala Lumpur Hospital, Jalan Pahang, 50586 Kuala Lumpur, Federal Territory, Malaysia

## Abstract

**Introduction:**

Basic research has indicated that tocotrienols have potent antiproliferative and proapoptotic effects that would be expected to reduce the effect of breast cancer.

**Methods:**

We conducted a double-blinded, placebo-controlled pilot trial to test the effectiveness of adjuvant tocotrienol therapy in combination with tamoxifen for 5 years in women with early breast cancer. Two-hundred-forty women, aged between 40 and 60 years, with either tumor node metastases (TNM) Stage I or II breast cancer and estrogen receptor (ER)-positive tumors were nonrandomly assigned to two groups. The intervention group received tocotrienol-rich fraction (TRF) plus tamoxifen, whereas the control group received placebo plus tamoxifen, for 5 years.

**Results:**

During the 5 years of the study, eight patients died of breast cancer, whereas in 36 patients, a local or systemic recurrence developed. Five-year breast cancer-specific survival was 98.3% (95% confidence interval (CI), 95.9% to 100%) in the intervention group and 95% (95% CI, 91.1% to 98.9%) in the control group, whereas the 5-year disease-free survival was 86.7% (95% CI, 80.6% to 92.8%) and 83.3% (95% CI, 76.6% to 90.0%), respectively. Risk of mortality due to breast cancer was 60% (HR, 0.40; 95% CI, 0.08 to 2.05) lower in the intervention group versus the controls after adjustment for age, ethnicity, stage, and lymph node status, but this was not statistically significant. Adjuvant TRF therapy was not associated with breast cancer recurrence (HR, 0.84; 95% CI, 0.43 to 1.65).

**Conclusions:**

From the current study, no association seems to exist between adjuvant tocotrienol therapy and breast cancer-specific survival in women with early breast cancer.

**Trial registration:**

ClinicalTrials.gov Identifier: NCT01157026.

## Introduction

Vitamin E is a potent antioxidant classified into subgroups of tocopherols and tocotrienols. In most food sources, tocopherols are more prevalent than tocotrienols. Palm oil is a particularly rich source of α-, γ-, and δ-tocotrienol [1,2]. Previous studies reported that individual tocopherols and tocotrienols exhibit different potencies in suppressing tumor cell growth and inducing apoptosis in neoplastic mammary epithelial cells [3,4]. Administrations of α- and γ-tocotrienol, and not α-tocopherol, have shown a life-prolonging effect in mice from transplanted tumors [5]. Tocotrienols also were found to suppress the growth of human breast and colorectal cancer cells [6-9], but α-tocopherol was not. These effects of tocotrienols may be explained through mechanisms such as antiangiogenesis, antiproliferation, induction of apoptosis, and improving immunologic functions [10].

Because tocotrienols are more potent than tocopherols [6-8,11,12], we hypothesize that tocotrienol intake may be associated with improved survival and lower recurrence in patients with early breast cancer. We investigated the effectiveness of TRF in combination with tamoxifen compared with placebo with tamoxifen as adjuvant therapy in improving breast cancer-specific survival in women with estrogen receptor (ER)-positive tumors who had been treated for TNM stage 1 and stage 2 (early) breast cancer. It should also be noted that tocotrienols with tamoxifen have shown a synergistic inhibitory effect on the proliferative rate and growth of breast cancer cells *in vitro *[11].

## Materials and methods

### Study design

We conducted a double-blinded, placebo-controlled trial for 5 years comparing tocotrienol-rich fraction (TRF) plus tamoxifen with placebo plus tamoxifen in women with early breast cancer. In total, 240 women with early breast cancer were assigned to two treatment groups by nonrandom allocation. The intervention group was given TRF plus tamoxifen (*n *= 120), whereas the control group was given placebo plus tamoxifen (*n *= 120). The primary end point was breast cancer-specific survival, defined as the time from allocation to treatment group to death of breast cancer. Breast cancer-specific survival was chosen as primary outcome based on overall health-protective effects conferred by the multiple functions of tocotrienols, including anticancer, anti-inflammatory, and antioxidant effects. The secondary end points included recurrence (either local or systemic), biochemical parameters, liver function, and plasma levels of vitamin E.

### Study population

In total, 240 patients treated in Kuala Lumpur Hospital, Malaysia, were recruited between November 2001 and November 2002. Only women with histologically confirmed primary breast cancer and estrogen receptor-positive tumors (determined with an immunohistochemistry test with a 10% cut-off point for positivity) were recruited into the study. Other criteria for eligibility included age of 40 to 60 years at the start of the tamoxifen therapy and an Eastern Cooperative Oncology Group performance status of 0, 1, or 2 (scored on a scale of 0 to 5, with lower scores indicating better function) [13]. Criteria for exclusion were the concurrent use of investigational drugs and estrogen-receptor status negative or unknown.

### Treatment program

Patients were blinded to the treatment that they received. Both the TRF and placebo drugs were prepared and supplied by Hovid Sdn. Bhd., Malaysia. The TRF drug was prepared in a capsule form at a dose of 200 mg per capsule. The placebo drug, which contained soy oil without tocotrienols, had an appearance and taste similar to that of the TRF drug. Patients were allocated to two treatment groups that received a daily dose of two capsules (one active TRF 200-mg capsule or placebo capsule, along with a tamoxifen 20-mg tablet). Patients were instructed to take the two capsules together at approximately the same time each day. Trial treatment continued for 5 years from the date of recruitment. Patients were assessed every 3 months for compliance by collection of bottles containing capsules and random visits to their homes by the research staff.

The assessors were also blinded to the treatment received by the trial patients. Baseline assessment included demographic and clinical profiles, such as age, race, breast-cancer stage, and involvement of lymph nodes. Clinical evaluations, routine blood tests for hematology, and blood chemistry assessment were performed every 6 months during year 1 and annually thereafter. Patients were monitored for compliance with follow-up, whereby those who defaulted were contacted and, when necessary, new appointment dates given. Patients underwent several biochemical screening tests, such as complete blood count (CBC), differential count, and liver-function tests. These biochemical parameters were also used to determine the health conditions of the patients. Mammography was performed annually throughout the study. Both local and systemic recurrence of disease was defined pathologically or on the basis of clinical or radiologic findings, and recurrences were dated at the time they were first detected. Additionally, all deaths recorded in this study were confirmed as due to breast cancer. The ethical clearance for this study was approved by the Research and Ethics Committee of the Ministry of Health, Malaysia (National Medical Research Register: Research Registration ID: 5399 S1). Written informed consent was obtained from all patients.

### Vitamin E extraction from blood plasma and HPLC analysis

Blood samples collected in heparinized tube were spun at 2,000 rpm for 10 minutes at room temperature. The plasma was isolated from the sedimented red blood cells and transferred into a sterile 1.5-ml centrifuge tube. After this, 500 μl of the plasma was then added to a tube containing 0.5 ml of 0.5% NaCl and ethanol. Then 400 μl of hexane was added into this tube. The mixture was shaken vigorously for an hour by using a mini-shaker. The tubes were then spun at 3,000 rpm for 10 minutes at room temperature. After centrifugation, the clear hexane phase was transferred carefully into a clean vial and blow-dried under nitrogen gas. An aliquot of the lipid sample was reconstituted in 500 μl hexane. Then 10 μl of the sample and a standard solution mixture of α-tocopherols, α-, γ-, and δ-tocotrienols was also injected accordingly into a HPLC system. The excitation wavelength and emission wavelength of the fluorescence detector were set at 295 and 325 nm, respectively. The mobile phase was hexane-isopropyl alcohol (99.5/0.5, vol/vol) with a flow rate of 2 ml/min. The peak areas of the components in the sample were compared with those of the standards and were used for quantitative calculation.

### Statistical analysis

The sample size was calculated under the assumption of a 5-year disease-specific survival of 95% in the tamoxifen-plus-placebo group and the detection of a difference of 5% in the 5-year disease-specific survival rate. These assumptions necessitated the enrollment of 240 women.

Categoric variables were described in proportion and compared by using the χ^2 ^test, whereas continuous variables were described in means and compared by using the *t *test. The analysis was based on the intention-to-treat principle. Kaplan-Meier analysis was used to estimate breast cancer-specific survival and disease-free survival, comparing the treatment versus the control group. Survival curves were compared by using the log-rank test. Cox regression analysis was used to compute crude hazard ratios to determine the association between TRF-plus-tamoxifen intake and breast cancer-specific death as well as recurrence of breast cancer, compared with the intake of placebo plus tamoxifen, which is the reference group. This model was subsequently adjusted for age (continuous), TNM stage (1 or 2), ethnicity (Malay, Chinese, Indian), and lymph node involvement (yes, no). Two-sided *P *values lower than 0.05 and HR with 95% confidence interval (CI) which does not include 1.00 were considered statistically significant. Data obtained during the study were processed by using SPSS for Windows (Version 18.0; SPSS Inc., Chicago, IL).

## Results

### Patient characteristics

**Figure 1:**
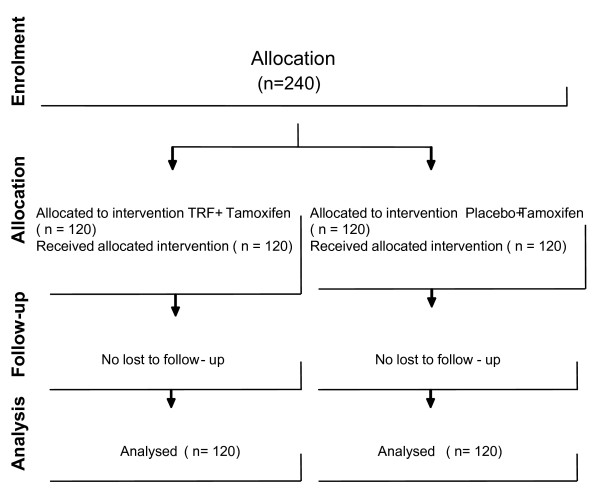
**Flow diagram of the progress through the phases of the parallel trials of two groups (enrolment, intervention allocation, follow-up, and data analysis)**.

The median duration of follow-up was 60 months, with complete follow-up of all patients who complied with their treatment throughout the study. Eight patients (two in the intervention group versus six in the control group) had died of breast cancer, and 36 patients (16 in the intervention group versus 20 in the control group) had local or systemic recurrence. Figure 1 shows the CONSORT diagram of the study. The groups were well balanced with respect to demographic and tumor characteristics (Table 1). Approximately 61% of patients in the TRF-plus-tamoxifen group and 49% of patients in the placebo-plus-tamoxifen group were in TNM stage 1 of breast cancer, whereas 39% of patients in the TRF-plus-tamoxifen group and 51% in the tamoxifen group were in TNM stage 2 (*P *= 0.07). A total of 58% of patients in the TRF-plus-tamoxifen group and 63% of patients in the placebo-plus-tamoxifen group showed involvement of a lymph node (*P *= 0.51). The average number of lymph nodes involved among these patients was 1.5 and 1.1, respectively (*P *= 0.15).

**Table 1:** Baseline demographic and tumor profile of 240 breast cancer patients with estrogen receptor-positive tumors

	Tocotrienol + tamoxifen (*n *= 120)	Tamoxifen only (*n *= 120)	*P *value
Age (years)	48.5 (5.2)	49.1 (5.9)	0.30
			
Ethnicity:			
Malay	50.0 (60)	46.7 (56)	0.61
Chinese	30.0 (36)	37.5 (45)	0.22
Indian	20.0 (24)	15.8 (19)	0.40
			
Stage of breast cancer:			0.07
1	60.8 (73)	49.2 (59)	
2	39.2 (47)	50.8 (61)	
			
Involvement of lymph node:			
Yes	58.3 (70)	62.5 (75)	0.51
Number of lymph nodes involved^a^	1.5 (3.1)	1.1 (1.9)	0.15

Continuous variables are expressed as means (standard deviations), and categoric variables, as percentages (numbers). ^a^Includes only patients with lymph node involvement.

**Figure 2:**
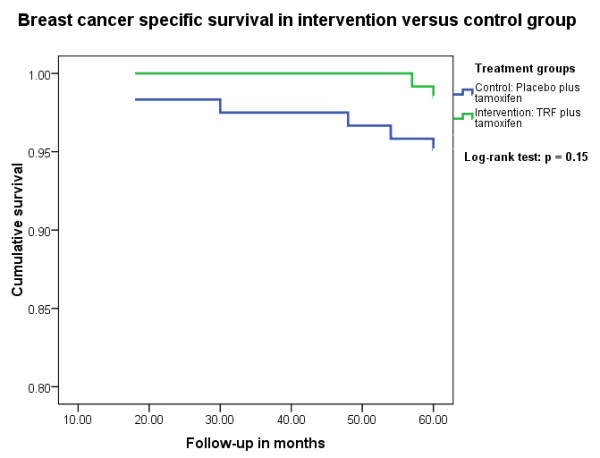
**Breast cancer-specific survival in intervention versus control group**.

Based on Kaplan-Meier analysis, 5-year breast cancer-specific survival in the TRF-plus-tamoxifen group was 98.3% (95% CI, 95.9% to 100%) compared with 95% (95% CI, 91.1% to 98.9%) in the placebo-plus-tamoxifen group (Figure 2). *P *for the log-rank test was 0.15. The 5-year recurrence-free survival was 86.7% (95% CI, 80.6% to 92.8%) and 83.3% (95% CI, 76.6% to 90.0%), respectively. *P *for log-rank test was 0.47.

The crude hazard ratio for breast cancer-specific death in the TRF-plus-tamoxifen group compared with placebo-plus-tamoxifen group was 0.33 (95% CI, 0.07 to 1.61) (Table 2). After adjustment for age, ethnicity, stage, and lymph node status, the multivariate HR was 0.40 (95% CI, 0.08 to 2.05) and statistically was not significant. Compared with placebo intake, intake of tocotrienol by patients with stage 1 or stage 2 breast cancer, and estrogen receptor-positive tumor treated with tamoxifen, is associated with a not statistically significant (*P *= 0.27) 60% lower risk of mortality, after adjustment for age, ethnicity, stage, and lymph node status.

**Table 2:** Tocotrienol intake and risk of breast cancer-specific death/recurrence in women with early breast cancer and estrogen receptor-positive tumors receiving tamoxifen

	Type of treatment received along with tamoxifen	
	
	Placebo (*n *= 120)	Tocotrienol (*n *= 120)
Breast cancer-related death		
Number of patients (*n *(%))	6 (5.0)	2 (1.7)
Crude hazard ratio	1.00	0.33 (95% CI, 0.07 to 1.61)
Adjusted hazard ratio^a^	1.00	0.40 (95% CI, 0.08 to 2.05)
		
Local/systemic recurrence of breast cancer		
Number of patients (*n *(%))	20 (16.7)	16 (13.3)
Crude hazard ratio	1.00	0.80 (95% CI, 0.41 to 1.54)
Adjusted hazard ratio^a^	1.00	0.84 (95% CI, 0.43 to 1.65)

^a^Adjusted for age (continuous), ethnicity (Malay, Chinese, Indian), TNM stage (stage 1, stage 2), and lymph node involvement (yes, no).

The multivariate HR for local or systemic recurrence in the TRF-plus-tamoxifen group compared with the placebo-plus-tamoxifen group was 0.84 (95% CI, 0.43 to 1.65).

### Biochemical parameters in study population

The mean values of the results from the biochemical screening tests on day 0, year 1, 2, 3, 4, and 5 of TRF-plus-tamoxifen supplemented group and placebo-plus-tamoxifen supplemented group are shown in Table 3.

**Table 3:** Blood parameters

Parameters		Range	Day 0	Year 1	Year 2	Year 3	Year 4	Year 5
	**Group**		**(Mean ± SD)**					
**White blood count**								
	Placebo + Tamoxifen	4-11	6.29 ± 2.09	6.77 ± 1.79	7.06 ± 2.07	7.11 ± 2.37	7.22 ± 7.22	7.36 ± 7.36
	TRF + Tamoxifen	(×10^9^/L)	5.86 ± 2.01	5.81 ± 1.59	5.86 ± 1.78	6.28 ± 1.92	6.33 ± 1.76	6.46 ± 1.62
								
**Red blood count**								
	Placebo + Tamoxifen	3.8-5.8	4.48 ± 0.48	4.46 ± 0.44	4.41 ± 0.40	4.49 ± 0.46	4.43 ± 0.54	4.55 ± 0.53
	TRF + Tamoxifen	(×10^12^/L)	3.95 ± 0.81	4.24 ± 0.41	4.27 ± 0.34	4.28 ± 0.34	4.27 ± 0.45	4.28 ± 0.40
								
**Hemoglobin**								
	Placebo + Tamoxifen	11.5-16.5	12.49 ± 1.28	12.40 ± 1.18	12.63 ± 1.16	12.66 ± 0.96	12.61 ± 0.92	12.70 ± 0.96
	TRF + Tamoxifen	(g/dl)	11.79 ± 2.48	12.71 ± 1.13	12.67 ± 0.93	12.72 ± 0.99	12.65 ± 1.27	12.77 ± 1.01
								
**Hematocrit**								
	Placebo + Tamoxifen	37-47	38.90 ± 3.27	38.34 ± 2.56	38.53 ± 2.82	38.48 ± 2.68	37.44 ± 8.69	38.69 ± 3.06
	TRF + Tamoxifen	(%)	35.51 ± 7.30	38.76 ± 4.31	37.96 ± 4.32	38.68 ± 2.97	38.36 ± 4.19	38.63 ± 3.27
								
**Lymphocyte**								
	Placebo + Tamoxifen	20-45	33.03 ± 6.62	33.37 ± 6.21	34.30 ± 8.13	35.19 ± 6.27	34.82 ± 8.32	35.48 ± 7.20
	TRF + Tamoxifen	(%)	31.42 ± 8.35	33.79 ± 9.41	33.22 ± 8.58	34.65 ± 8.68	34.59 ± 6.63	36.39 ± 9.58
								
**Platelet count**								
	Placebo + Tamoxifen	150-400	221.52 ± 60.59	221.48 ± 46.09	222.19 ± 61.39	238.10 ± 61.63	250.00 ± 61.87	260.10 ± 57.85
	TRF + Tamoxifen	(×10^9^/L)	221.16 ± 89.81	217.89 ± 50.74	217.63 ± 58.52	226.13 ± 49.69	247.74 ± 76.05	243.07 ± 61.02

The mean values of the results from the biochemical screening tests on day 0 and years 1, 2, 3, 4, and 5 of tocotrienol-rich fraction (TRF)-plus-tamoxifen supplemented group and placebo-plus-tamoxifen supplemented group.

The biochemical parameters measured did not exhibit any significant changes from the results obtained before the start of the study for both TRF-plus-tamoxifen and placebo-plus-tamoxifen groups.

### Liver-function tests

The purpose of liver-function tests is to study the tolerance of the supplement. The mean values of the results from the liver-function tests on day 0, year 1, 2, 3, 4, and 5 of TRF-plus-tamoxifen supplemented group and placebo-plus-tamoxifen supplemented group are shown in Table 4.

**Table 4:** Liver-function tests

Parameters		Range	Day 0	Year 1	Year 2	Year 3	Year 4	Year 5
	**Group**		**(Mean ± SD)**					
**Total protein**								
	Placebo + Tamoxifen	66-87	76.68 ± 5.04	76.72 ± 5.89	77.56 ± 5.12	77.32 ± 4.55	75.48 ± 5.28	77.08 ± 5.10
	TRF + Tamoxifen	g/L	76.03 ± 4.71	76.31 ± 5.33	76.41 ± 5.07	76.82 ± 4.55	77.08 ± 4.65	76.54 ± 4.82
								
**Albumin**								
	Placebo + Tamoxifen.	35-50	41.48 ± 3.78	41.72 ± 3.80	40.84 ± 7.14	42.60 ± 4.29	41.48 ± 3.81	41.40 ± 7.46
	TRF + Tamoxifen	g/L	41.28 ± 3.24	41.36 ± 3.69	41.05 ± 3.64	42.00 ± 4.13	41.69 ± 4.46	41.92 ± 4.07
								
**Total bilirubin**								
	Placebo + Tamoxifen	< 21	7.80 ± 3.81	7.80 ± 4.17	8.40 ± 3.58	9.16 ± 3.98	8.12 ± 3.70	8.72 ± 4.09
	TRF + Tamoxifen	μ*M*	8.00 ± 3.94	7.74 ± 3.70	7.74 ± 3.88	8.15 ± 4.63	7.95 ± 3.72	8.16 ± 3.89
								
**Alkaline phosphatase**								
	Placebo + Tamoxifen	42-98	65.08 ± 17.09	63.88 ± 15.21	64.56 ± 19.58	66.80 ± 17.14	62.12 ± 15.14	65.28 ± 18.92
	TRF + Tamoxifen	U/L	66.67 ± 18.55	62.51 ± 20.15	66.18 ± 20.41	64.74 ± 18.44	67.79 ± 20.06	68.42 ± 19.92
								
**Alanine transaminase**								
	Placebo + Tamoxifen	< 32	28.28 ± 22.69	34.04 ± 50.06	23.28 ± 13.87	27.72 ± 25.74	32.28 ± 48.75	24.28 ± 18.16
	TRF + Tamoxifen	U/L	32.18 ± 29.63	32.85 ± 18.93	31.69 ± 20.55	32.13 ± 28.04	29.77 ± 15.25	28.79 ± 15.14
								

The mean values of the results from the liver-function tests on day 0 and years 1, 2, 3, 4, and 5 of tocotrienol-rich fraction (TRF)-plus-tamoxifen supplemented group and placebo-plus-tamoxifen supplemented group.

Liver-function tests (total protein, albumin, and total bilirubin) showed insignificant values for before the start and at completion of the study for the TRF-plus-tamoxifen supplemented group and the placebo-plus-tamoxifen supplemented group. Liver-function tests such as alkaline phosphatase and alanine transaminase showed high standard-deviation (SD) values. This is due to the collection of samples from patients after the chemotherapy session.

### High levels of vitamin E in plasma of patients supplemented with TRF

**Figure 3:**
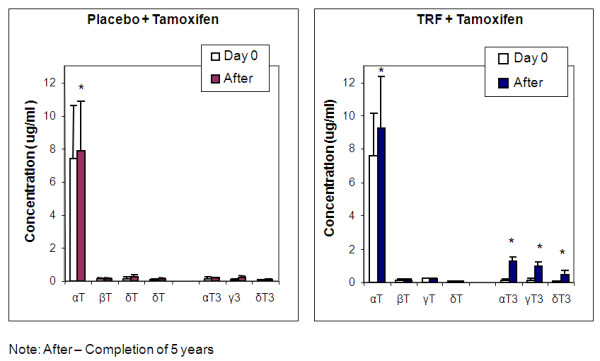
**The α-tocopherol, α-, γ-, and δ-tocotrienol concentrations increased significantly after 5 years for the TRF-supplemented group**. *P < 0.05.

Plasma samples obtained from patients were analyzed by using HPLC to quantify the concentrations of vitamin E in plasma (Figure 3).

The amount of endogenous α-tocopherol in the blood of patients from both groups increased significantly (*P *< 0.05) after the 5-year period compared with day 0 (Figure 3). The α-tocopherol, α-, γ-, and δ-tocotrienol concentrations increased significantly (*P *< 0.05) in patients who received TRF plus tamoxifen as compared with placebo plus tamoxifen (Figure 3).

However, the concentrations of tocotrienols in the placebo group after the 5-year period remained the same, and the amounts did not differ significantly (*P *> 0.05) as compared with those at day 0.

## Discussion

In our current pilot study, we found no protective association between tocotrienol intake and breast cancer-related mortality or recurrence in patients with early breast cancer. Tamoxifen-alone and TRF-plus-tamoxifen supplementation also did not change the blood parameters of patients before the start and upon completion of the study.

The risk of dying of breast cancer seems to be reduced by approximately 60% in patients receiving a combination of tocotrienol and tamoxifen compared with patients receiving placebo and tamoxifen. However, this was not statistically significant (*P *= 0.27). Previous studies carried out in our laboratory showed that tocotrienols inhibited growth and proliferation of breast cancer cells [6-8]. Another study reported that the anticarcinogenic property of tocotrienols in synergy with tamoxifen is more potent than tamoxifen alone [11]. Recently we also observed a novel mechanism by which tocotrienols inhibit breast cancer cell growth, in which the effects are mediated by binding to the ERβ and inducing the expression of specific genes containing estrogen-responsive element (ERE) sequences in their promoter [14].

Our results showed that the TRF-plus-tamoxifen supplemented group had significantly higher concentrations of α-tocopherol and α-, γ-, and δ-tocotrienols in the plasma of the breast cancer patients. Plasma α-tocopherol levels were much higher than those of any other tocotrienol isomers. This result supports the findings reported by [15], who reasoned that α-tocopherol transfer protein (α-TTP), together with the tocopherol-associated proteins (TAP) are responsible for the endogenous accumulation of natural α-tocopherol.

Concentrations of the micronutrient in blood plasma revealed that α-tocotrienols had the highest absorption, followed by γ- and δ-tocotrienols, in all individuals supplemented with tamoxifen and TRF at the end of study, as compared with placebo and basal concentrations of the micronutrients. Although the α-TTP that binds the vitamin E isoforms via low-density lipoprotein (LDL) have a much lower capacity to transport tocotrienols, it has been previously reported that orally supplemented tocotrienol results in plasma tocotrienol concentration in the range of 3 μ*M *[16].

In our study, liver function was not altered by the supplementation of tamoxifen in combination with TRF. It seems that this combination is well tolerated.

The major strength of this study is that it is the first experimental study that was conducted to investigate the effect of TRF on survival of patients with early breast cancer. Furthermore, we had a complete follow-up of all of our patients at every point of the assessment, and they also complied well with the treatment assigned to them.

The lack of randomization is a limitation in this study. However, the two groups were balanced with respect to demographic and tumor characteristics. In addition, a notable weakness in this study is that we had a relatively small sample size with a low number of outcomes, possibly resulting from using breast cancer-specific death as our primary outcome, leading to a low power to detect statistically significant differences in the outcomes between the two groups.

## Conclusions

Results from this study are not sufficient to indicate a significant association between adjuvant tocotrienol therapy and breast cancer survival in women with early breast cancer. It is important to note that accruing evidence suggests that tocotrienols have anticancer effects. Moreover, tamoxifen and tocotrienol *in vitro *have demonstrated synergy. Although a 60% lower mortality occurred in the intervention group, this result was not statistically significant. Hence, a large randomized trial is certainly warranted in the near future to establish whether tocotrienol adjuvant therapy can significantly improve recurrence or mortality or both.

## Abbreviations

α-TTP: α-tocopherol transfer protein; CBC: complete blood count; ER: estrogen receptor; HPLC: high-performance liquid chromatography; LDL: low-density lipoprotein; RBC: red blood cell; TAP: tocopherol-associated protein; TNM: tumor node metastases; TRF: tocotrienol-rich fraction.

## Competing interests

The authors declare that they have no competing interest. Hovid Sdn. Bhd. absolutely did not have any influence in the trial designing, patient recruitment, data collection, analysis, and reporting.

## Authors' contributions

The authors' responsibilities were as follows: KN, KRS, PAG, and GAR: study design, data collection, statistical analyses, and interpretation of data; and KN, KRS, PAG, GAR, and SDV: manuscript writing.
